# Oncolytic Virotherapy versus Cancer Stem Cells: A Review of Approaches and Mechanisms

**DOI:** 10.3390/cancers10040124

**Published:** 2018-04-19

**Authors:** Shyambabu Chaurasiya, Nanhai G. Chen, Susanne G. Warner

**Affiliations:** 1 Department of Surgery, Division of Surgical Oncology, City of Hope National Medical Center, Duarte, CA 91010, USA; schaurasiya@coh.org (S.C.); nchen@coh.org (N.G.C.); 2 Center for Gene Therapy, Department of Hematologic and Hematopoietic Cell Transplantation, Beckman Research Institute, City of Hope National Medical Center, Duarte, CA 91010, USA; 3 Gene Editing and Viral Vector Core, Beckman Research Institute, City of Hope National Medical Center, Duarte, CA 91010, USA

**Keywords:** oncolytic virus, cancer stem cells, viral therapy

## Abstract

A growing body of evidence suggests that a subset of cells within tumors are resistant to conventional treatment modalities and may be responsible for disease recurrence. These cells are called cancer stem cells (CSC), which share properties with normal stem cells including self-renewal, pluripotency, drug resistance, and the ability to maintain quiescence. While most conventional therapies can efficiently destroy rapidly dividing cancer cells comprising the bulk of a tumor, they often fail to kill the less abundant and quiescent CSCs. Furthermore, killing of only differentiated cells in the tumor may actually allow for enrichment of CSCs and thereby portend a bad prognosis. Therefore, targeting of CSCs is important to achieve long-term success in cancer therapy. Oncolytic viruses represent a completely different class of therapeutics that can kill cancer cells in a variety of ways, which differ from those of conventional therapies. Hence, CSCs that are inherently resistant to conventional therapies may be susceptible to oncolytic virus-mediated killing. Recent studies have shown that oncolytic viruses can efficiently kill CSCs in many types of cancer. Here, we discuss the mechanism through which CSCs can escape conventional therapies and how they may still be susceptible to different classes of oncolytic viruses. Furthermore, we provide a summary of recent studies that have tested oncolytic viruses on CSCs of different origins and discuss possible future directions for this fascinating subset of oncolytic virus research.

## 1. Introduction

Cancer cells are distinct from normal cells in that their growth is dictated by six characteristic changes in their physiology known as the hallmarks of cancer. These are self-sufficient growth signaling, insensitivity to anti-growth signals, resistance to apoptosis, immortality, sustained angiogenesis, tissue invasion, and metastasis [1]. These cellular changes arise from mutations in the underlying genetic sequences and/or epigenetic alterations [2]. One theory of cancer development is that of monoclonal origin, meaning that all cells in a tumor can be traced back to a single progenitor cell [3]. The clonal nature of tumors is a result of sequential accumulation of genetic and/or epigenetic changes. However, tumor development also depends on the tumor microenvironment.

To explain the observed properties of tumors, two models have been proposed: the “clonal evolution model” and the “cancer stem cell (CSC) model”. Both of these models can explain all the observed tumor properties but in different ways. However, neither of these models is clear regarding the type of cell that initiates cancer. Whether cancer originates from stem cells, progenitor cells, or terminally differentiated cells is still a subject of debate. While the clonal model assumes that any cell can become a cancer-initiating cell if it can accumulate transforming mutations, the CSC model assumes that normal stem cells are the most suitable candidates for accumulating transforming mutations [4]. The CSC model proposes that tumors originate from cells sharing many properties of stem cells including self-renewal, pluripotency, drug resistance and the ability to remain quiescent for a long period [5,6]. According to this model, CSCs reside at the top of the hierarchy and differentiate into non-stem populations in a unidirectional way, thus creating tumor heterogeneity [7]. If a tumor were to be compared with a beehive, CSC would be analogous to the queen bee. Therefore, in order to eliminate propagation of cancer cells, the queen bee should be targeted.

## 2. Identification of Cancer Stem Cells

The first study to provide evidence for the presence of CSCs came from the study of acute myeloid leukemia (AML) cells in 1994 [8]. In this study, hematopoietic cells were extracted from patients suffering from AML and injected into severe combined immune-deficient (SCID) mice with the expectation of modeling leukemic disease in vivo. The study showed that only a group of cells defined by CD34^+^CD38^−^, sharing the surface marker profile of normal immature multipotent progenitors [8,9], were able to generate leukemia in SCID mice. Interestingly, CD34^+^CD38^−^ cells were able to reconstitute the phenotypic heterogeneity observed in the original donor [8]. From these observations, it could be concluded that cancer cell populations are organized in a functional hierarchy much like that of a stem cell system [9].

In the context of solid tumors, the presence of CSCs was first demonstrated in breast tumors by Al-Hajj and colleagues in 2003 [10]. They showed that a subset of breast cancer cells expressing CD44^+^CD24^−/low^ were tumorigenic with as few as 10^2^ cells capable of initiating tumor growth in NOD/SCID mice. Upon serial passaging in mice, this subset of cells was able to generate new tumors with both CD44^+^CD24^−/low^ populations as well as phenotypically diverse populations of non-tumorigenic cells, recapitulating the complexity of initial tumor [10]. Since then, different surface markers have been used to isolate CSCs from various malignancies. However, the frequency of surface marker-based CSCs differ even within the same type of tumor [11]. Besides cell surface markers, properties of stem cells such as high activities of drug efflux pumps have also been used to isolate cancer stem-like cells [12]. For example, cells with the ability to exclude Hoechst 33342 dye, called the “side population” based on their staining pattern during fluorescent-activated cell sorting, have been isolated from breast and other cancers. This population has been found to be enriched in cells with CSC properties such as self-renewal and high tumorigenicity [12,13]. Other functional assays such as tumorsphere formation and aldehyde dehydrogenase (ALDH) expression have also been used to identify CSCs in solid tumors as well. Indeed, researchers have isolated CSC populations from many different solid tumors including brain, lung, liver, melanoma, colorectal, sarcoma, head and neck, pituitary, and pancreatic cancer [14].

## 3. Conventional Cancer Therapy and CSCs

Despite advancements in radiation and chemotherapy, complete tumor regression is rare. Although most patients show initial response to the treatment, development of resistance during the course of treatment and tumor recurrences are common. Many traditional cancer therapies rely on apoptosis induction to kill cancer cells [15]. As such, these interventions are not effective against apoptosis-resistant cancer cells, including CSCs [16]. Moreover, CSCs and normal stem cells express protective drug transporters such as multi-drug resistance transporter and ATP binding cassette (ABC), which can pump anticancer (cytotoxic) chemicals out of the cells [16,17]. This may further explain the inherent resistance of CSCs to conventional chemotherapy [18,19]. Likewise, CSCs are also resistant to radiation therapy, which some investigators have attributed to their increased defense against reactive oxygen species as a result of hypoxia [20], or their elevated level of DNA repair mechanisms [21]. Furthermore, anticancer agents mainly kill rapidly dividing cells, but CSCs can remain in a quiescent state for a prolonged period of time. For instance, in leukemia CSCs mainly exist in G0 (non-dividing) phase and are inherently resistant to chemotherapeutics [22]. While the current therapies exploit the genetic differences between cancer cells and normal cells, they fail to consider the differences in gene expression of different cells within individual tumors [22]. Conventional therapies, which often target highly proliferating cells, are prone to miss the CSCs that are slow dividing and can maintain quiescence for a long time [23,24,25]. Furthermore, killing of only differentiated cells in the tumor may allow for enrichment of CSCs that can cause disease recurrence with worse prognosis [26]. Therefore, targeting of CSCs is important to achieve long-term success in cancer therapy. As in tumors in vivo, a subset of cells in cancer cell lines in vitro appears to have CSC phenotypes, allowing candidate therapeutics to be tested in vitro on CSCs without having to isolate CSCs from animals/humans [11]. It is for this reason that most of the novel therapeutics are tested for their effect on CSCs in preclinical and/or clinical trials.

## 4. Oncolytic Viruses and Mechanisms of Cancer Cell Death

Oncolytic viruses (OVs) infect, replicate in, and kill malignant cells. The properties of cancer cells that allow them to thrive also make them better hosts for many types of viruses. For example, cancer cells often have non-functional innate immune defense mechanisms as a consequence of their need to evade detection and destruction by the immune system. Cancer cells with defective interferon pathways are highly permissive to vesicular stomatitis virus, myxoma virus and raccoonpox virus, which are otherwise attenuated in normal cells [27,28,29]. Also, cancer cells tend to resist apoptosis and translational suppression, both of which are favorable for the growth of several types of viruses [30]. Furthermore, over-expression of certain virus-receptors by cancer cells allow higher uptake of viruses compared to normal cells. For example, several types of cancer cells over-express CAR [31], laminin [32], CD155 [33] and CD46 [34] which allow for higher uptake of adenovirus [35], sindbis virus [36], polio virus [37] and measles virus [38], respectively.

Oncolytic viruses are thought to exert their antineoplastic effects via multiple different pathways. While the exact mechanism of oncolysis differs from virus to virus; there are some common mechanisms employed by most oncolytic viruses to achieve an antineoplastic effect. First, replication of viruses in cancer cells can induce lysis of the cell [39]. However, cell death caused by the direct replication of oncolytic viruses is complex and the conventional concepts of cell death such as apoptosis, necrosis and autophagy are generally inadequate to fully describe such cell death [30]. This is partly because oncolytic viruses are thought to hijack the cell death machinery, allowing death to occur only when cellular resources have been fully exploited for maximal production of progeny viruses [30]. Oncolytic viruses are self-perpetuating and a single dose of the virus, in theory, could eliminate all cancer cells unless the virus itself is cleared by the immune system before cancer cells are eliminated [40].

Second, some viral proteins are toxic to cancer cells, which can directly kill the cell before replication-mediated lysis. For example, the 11.6 kDa E3 death protein and E4orf4 proteins encoded by adenovirus are toxic to cells and cause cell death upon exposure^240,241^. This type of cell death by oncolytic viruses is undesired since premature cell death prevents proliferation and optimal release of oncolytic viruses from infected cells for subsequent rounds of infections.

Third, oncolytic viruses could induce specific and non-specific anti-tumor immunity, which can enhance the overall efficacy of the virus. Although the role of the immune system has been a matter of debate for a long time in oncolytic virotherapy, recent advancements suggest that the immune system plays a favorable role [41]. Moreover, oncolytic viruses are often engineered to encode a therapeutic gene that can further aid to the overall anti-tumor efficacy of the virus. A variety of transgenes ranging from immune-stimulatory genes to pro-apoptotic genes have been inserted into different oncolytic viruses to enhance their anti-tumor efficacy. For example, the immune-stimulatory genes IL-2, IL-4, IL-12 and GM-CSF as well as pro-apoptotic genes such as tumor necrosis factor alpha, p53 and tumor necrosis factor-related apoptosis inducing ligand (TRAIL) have been studied as therapeutic genes in different oncolytic viruses [42,43,44,45,46,47,48].

Lastly, recent studies have shown that oncolytic viruses can indirectly kill tumor cells by destroying tumor vasculature [49,50]. Adequate blood supply within the tumor is a critical requirement for tumor progression and metastasis. Therefore, inhibition of angiogenesis and/or disruption of established tumor vasculature could potentially result in tumor regression.

A diverse array of virus families have been studied for their oncolytic potential including *Rhabdoviridae*, *Poxviridae*, *Herpesviridae*, *Reoviridae*, *Adenoviridae*, *Paramyxoviridae*, *Picornaviridae*, *Togaviridae,* and *Parvoviridae* (reviewed by Vaha-Koskela [51]). Some of these oncolytic viruses have been tested for their potential to target and kill CSCs in different types of cancer (Table 1), which will be discussed in detail below.

## 5. Mechanisms of Resistance to Chemotherapy and Radiation Therapies and the Prospect of Oncolytic Viruses in Killing CSCs

Much like normal stem cells, CSCs reside in niches. A CSC niche is defined as a specialized microenvironment where CSCs are located and where they can interact with other types of cells including immune cells, endothelial cells, fibroblasts and peri-vascular cells as well as secreted factors such as cytokines, growth factors and extracellular matrix (ECM) [66]. Different components of the CSC niche play a role in protecting CSCs from therapeutic interventions. CSCs have been shown to over-express different types of integrins, which allow them to be drug resistant [67]. Integrins are cell surface glycoproteins that serve as receptors for ECM and other cellular as well as soluble ligands. Indeed, integrin expression is required for the acquisition of CSC characteristics and for metastasis [67]. CSC niche-associated resistance to therapy depends on the adhesion of CSCs to ECM via integrins [68]. While integrins can make CSCs resistant to chemo and radiation therapies, the same integrins could be used to specifically target CSCs with oncolytic virus. For example, Jiang et al. showed that an oncolytic adenovirus that has an RGD (Arg-Gly-Asp) peptide inserted into its H1-loop, enters into tumor cells via integrins and the virus was shown to be effective against CSCs isolated from patients with brain tumor via serum-free culture methods [59]. CSCs were found to express integrins as well as the natural receptor for adenovirus i.e., coxsackie-adenovirus receptors. A similar oncolytic adenovirus with RGD-modified capsid was shown to be very effective against bladder CSC-like cancer initiating cells identified via cells cultured as spheroid expressing multi-drug resistance protein, Nanog, and surviving [69]. While not specifically singling out CSCs, Ammayapan et al. similarly inserted cyclic RGD into the envelope glycoprotein of vasicular stomatistis virus (VSV) for transductional targeting of the virus to cells over-expressing multiple types of integrins [70]. This virus was found to target integrin over-expressing cells both in vitro and in murine models.

Recent studies have shown that CSCs play a role in tumor angiogenesis [71]. Furthermore, CSCs have been shown to form vessel-like channels through a process called vascular mimicry (VM) [72,73]. CSCs are thought to reside in a complex microvascular niche, encompassing VM as well as authentic blood vessels formed of endothelial cells [73]. Similar to tumor vasculatures made of endothelial cells, the VM also serve as a channel for nutrient supply and an alternative access point for metastasis [73,74]. The microvascular niche is important for the survival of CSCs, which is evident from the study published by Folkins et al. wherein a targeted anti-angiogenic therapy resulted in loss of stemness in brain CSCs and sensitized them to chemotherapy [75]. Interestingly, oncolytic viruses can induce profound and rapid collapse of established tumor vasculature and they can also block formation of new blood vessels by inhibiting vascular endothelial growth factor [76]. Extrapolating from these findings in non-CSC arenas, one could hypothesize that by inhibiting vascular niche, oncolytic viruses can make CSCs susceptible to virus-mediated killing and other therapeutics.

Furthermore, Notch signaling plays an important role in connection of angiogenesis and self-renewal of CSCs and has been shown to make CSCs resistant to chemo and radiation therapies [77,78]. Notch is an example of a pathway that, while conferring CSCs resistance to conventional therapies, may also be helpful in targeting of CSCs with oncolytic virus. For example, Mato-Berciano et al. constructed an oncolytic adenovirus in which they used a chimeric promoter with Notch-responsive element to control the essential viral gene E1A [79]. This virus was shown to effectively kill both differentiated cancer cells and pancreatic CSCs in vitro and showed a strong anti-tumor activity against pancreatic tumors in murine models leading to reduction in the ability of treated tumors to form tumorspheres.

**Figure 1:**
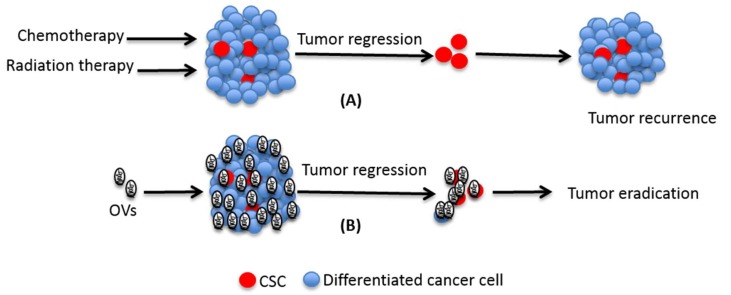
Role of cancer stem cell (CSCs) in traditional treatment failure. While chemo- and radiation-therapy can efficiently kill differentiated cells that make the bulk of tumor, they often fail to kill CSCs. Due to their ability to undergo self-renewal and differentiation, the surviving CSCs can cause disease relapse after initial remission. Unlike the conventional therapies, OVs have the potential to kill both differentiated cells as well as CSCs and hence they may cause eradication of the disease.

Although the initial CSC hypothesis postulates that CSCs reside at the top of the hierarchy and differentiate into non-CSC populations in a unidirectional way, recent studies have put forth compelling evidence that non-CSCs retain the ability of reverting to CSC-like state [80,81,82,83]. In different types of malignancies, residual tumors following therapy have been found enriched in CSC-like cells [81,84]. Previously the enrichment of CSCs was thought to be solely due to selective killing of differentiated non-CSCs as they are more susceptible to conventional therapies compared to the CSCs. However, only recently investigators have realized that cellular plasticity plays a role in controlling the stemness of cells and, that the observed increase in the proportion of CSC-like cells post-therapy is not merely a result of specific killing of non-CSCs. That cellular plasticity contributes to the increase in CSC-like cells post-therapy was categorically confirmed through lineage tracing analysis at single cell level in glioma cells after treating with temozolomide [84]. One of the major events leading to the conversion of non-CSC to CSC is epithelial to mesenchymal transition (EMT) [85,86]. Cancer cells through the process of EMT acquire invasive and metastatic properties and resistance to traditional therapies [87]. In contrast to the effect of EMT on traditional therapies, Chen et al. have shown that EMT may enhance the response to an oncolytic herpes virus [88]. In line with this, Marcato et al. found that an oncolytic reovirus can efficiently kill CSCs in vitro and, unlike traditional therapies, does not increase the proportion of CSCs in tumor xenografts [57]. Taken together, many components of the tumor microenvironment that make CSCs resistant to conventional therapies, are ill-equipped to confer that same resistance to oncolytic viruses (Figure 1).

Radiation and many chemotherapeutic agents kill cancer cells by inducing DNA damage [89]. In response to DNA damage, cancer cells tend to stop proliferation through the activation of the checkpoint kinases 1 and 2 (ChK1/2) and ataxia telangiectasia mutated (ATM) to allow DNA repair [21]. While all types of cells have the ability to repair their DNA following genotoxic insult, CSCs repair their damaged DNA more rapidly than non-CSCs. In fact, CSCs display basal activation of chk1/2 and ATM, suggesting that these cells are primed to respond to genotoxic insults [90]. Accordingly, multiple studies have shown that CSCs from different tumor types are notoriously resistant to radiation and genotoxic drugs. Interestingly, oncolytic viruses, DNA viruses in particular, have been found to either block DNA repair or take advantage of the DNA damage response for their own replication. For example, adenovirus has been shown to block DNA damage repair by dampening double strand damage sensing through interference with the activity of MRE11-Rad50-Nbs1 complex, and by inhibition of DNA ligase IV, a key enzyme involved in the final step of DNA healing in the non-homologous end joining (NHEJ) process. In line with this, several studies have shown that oncolytic adenovirus can sensitize cancer cells to genotoxic drugs [91,92]. Likewise, the process of DNA damage repair has been found to enhance the replication of oncolytic herpes virus and the resulting oncolysis thereof [93,94]. Therefore, enhanced DNA damage response has no negative effect on the efficacy of oncolytic viruses; in fact, DNA damage response may either make CSCs more susceptible to oncolytic virus or oncolytic virus-mediated sensitization to genotoxic drugs.

**Figure 2:**
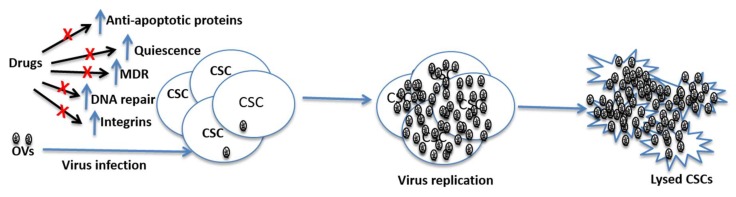
Potential mechanisms through which CSCs can resist conventional therapies, and prospects of OVs in killing such cells. CSCs have high levels of MDR expression, increased DNA repair ability and they can maintain quiescence for a long time. These features of CSCs can render chemotherapeutic drugs ineffective in killing them. However, oncolytic viruses are not affected by these features and hence they can replicate in CSCs leading the lysis of these cells. Note: OVs = Oncolytic viruses; MDR = multi-rug resistance gene; CSCs = cancer stem cells.

Adequate blood supply within the tumor is a critical requirement for tumor progression and metastasis. Therefore, effective inhibition of angiogenesis and/or disruption of established tumor vasculature could potentially result in tumor regression. To harness the therapeutic benefit by inhibiting tumor vasculature several anti-angiogenic drugs such as bevacizumab and sorafenib have been approved while many others are currently being evaluated in different phases of clinical trials [95,96]. However, the benefits from anti-angiogenic drugs have been modest, at best [97]. One drawback of anti-angiogenic drugs is that they do not directly kill cancer cells, and hence they are unlikely to eradicate tumors on their own. Furthermore, hypoxia has been shown to promote stem-like properties in cancer cells [66]. It has long been known that compared to normoxic cells hypoxic cells are more resistant to both chemo and radiation therapies [98,99]. Thus, hypoxia in tumor adds another hurdle in the targeting of CSCs. As discussed above, anti-angiogenic/anti-vasculature is a common property shared by many oncolytic viruses [100,101,102]. However, unlike anti-angiogenic drugs, OVs can directly kill both cancer cells and endothelial cells. Furthermore, while chemo and radiation therapies are severely affected by hypoxia, oncolytic viruses seem to be less prone to hypoxia-related hindrances. Indeed, hypoxia may enhance the anti-tumor efficacy of some oncolytic viruses. For example, hypoxia has been found to increase replication efficiency of herpes simplex virus, which is thought to result from increased MEK activity in hypoxic cells [103]. Likewise, hypoxia has been reported to enhance the cytotoxic ability of vaccinia virus without affecting virus replication [104,105]. Furthermore, oncolytic viruses could be modified to utilize hypoxic condition to enhance their growth and cytotoxic ability. For instance, Reinblatt et al. inserted hypoxia-responsive sequences upstream of ribonucleotide reductase gene in herpes simplex virus and by doing so, they were able to enhance the oncolytic activity of this virus in hypoxic tumor cells [106]. Thus, as schematically summarized in Figure 2, oncolytic viruses seem better suited to combat CSCs by using the very mechanisms that they have exploited to evade conventional therapies.

## 6. Oncolytic Viruses Targeting CSCs

### 6.1. Leukemia

CSCs in hematological malignancies have been shown to be resistant to chemotherapy and radiation therapy [107,108,109] but appear to be as sensitive to oncolytic viruses as the non-CSC population. Tong et al. showed that their oncolytic adenovirus encoding Beclin-1, an autophagy inducing protein, could efficiently kill leukemic CSCs in vitro. Becklin-1 expression was found to induce autophagy both in CSC and non-CSC populations in vitro and the oncolytic virus was able to completely eliminate established xenografts in mice [52]. Furthermore, the oncolytic virus was found to significantly reduce colony-forming ability of primary CML cells isolated from human patients with imatinib-resistant disease. Perhaps the best evidence for selective killing of leukemic stem cells by an oncolytic virus comes from a study by Kim et al., which showed that an oncolytic myxoma virus could efficiently kill leukemia stem cells and progenitor cells while leaving normal hematopoietic stem cells unharmed [98]. In this study, the authors demonstrated that oncolytic myxoma virus could be successfully used for purging leukemic stem cells before autologous transfer of blood and marrow which is used in the treatment of certain cancers that are refractory to standard therapies [98].

### 6.2. Breast Cancer

In 2007, Eriksson et al. isolated CSCs (CD44^+^CD24^−/low^) from pleural effusions of breast cancer patients and tested two oncolytic adenoviruses: Ad5/3-Δ24 and Ad5.pk7-Δ24. Both the oncolytic viruses were found to potently kill the CSCs in vitro and infection of the CSCs with either of the two viruses abrogated tumor-forming capability of those CSCs in SCID mice [54]. Another study from the same group evaluated the potency of transcriptionally-targeted oncolytic adenoviruses in killing CSCs isolated from pleural effusions of breast cancer patients. They constructed different oncolytic adenoviruses each of which had an essential gene (E1A) under the control of tumor-specific promoters: human telomerase reverse transcriptase (hTERT), cyclo-oxygenase-2 (Cox-2), multi-drug resistant (MDR) and alpha-lactalbumin [55]. Among these oncolytic viruses, Cox-2 and MDR-regulated viruses were found to be very effective against CSCs. These two viruses were able to kill the CSCs in vitro and showed significant anti-tumor effect against CD44^+^CD24^−/low^–derived xenografts. The authors concluded that high activity of the Cox-2 and MDR promoters in CD44^+^CD24^−/low^ cells was responsible for high oncolytic efficacy of the viruses [55]. Moreover, Li et al. have shown that an oncolytic herpes simplex virus deleted of *γ34.5* could potently kill the CD44^+^CD24^−/low^ population isolated from human breast cancer cell line SKBR-3 as well as primary human breast cancer cells [56]. At very low dose, the virus was found to be highly cytotoxic in vitro, and in murine models the virus showed significant anti-tumor effect against tumors derived from these cells. Likewise, Marcato et al. have shown that an oncolytic reovirus could kill both CSCs and non-CSCs equally, both in vitro and in vivo in mouse models [57]. The levels of Ras, which determines oncolytic activity of reovirus, was found to be similar in CSC and non-CSC populations. Wang et al. found that an oncolytic vaccinia virus (GLV-1h68) lacking 3 genes (*J2R*, *F14.5* and *A56R*) replicated more efficiently in CSCs compared to non-CSCs isolated from a human breast cancer cell line GI-101 [110]. The virus was able to eradicate tumors originating from CSCs in mice. In this study, the authors considered ALDH positive CD44^+^CD24^+^ cells as CSCs. Furthermore, we have found that an oncolytic vaccinia virus lacking the *F4L*, the small subunit of ribonucleotide reductase, could efficiently kill CSCs isolated from the inflammatory triple-negative breast cancer cell line SUM-149 [111]. Of note, inflammatory triple-negative breast cancer represents the most aggressive type of breast cancer and the CSCs populations from SUM-149 have been shown to be notoriously resistant to chemotherapeutics [112,113,114].

### 6.3. Glioblastoma

In the context of brain cancer, cells with surface expression of CD133 have the ability of self-renewal and differentiation and hence are considered as CSCs. Jiang et al. for the first time studied feasibility of an oncolytic virus in killing CSCs in brain cancer [59]. In their study, the authors isolated CSCs from 4 fresh glioblastoma specimens obtained from patients and tested the oncolytic activity of an engineered adenovirus Delta-24-RGD that could replicate in cells with defective retinoblastoma protein (Rb) [59]. The CSCs were found to express high levels of virus receptors on their surface and had defective Rb pathway. Consequently, the CSCs were found to support high levels of virus infection, replication and oncolysis. The infected cells mostly died via autophagy as evident from accumulation Atg5, LC3-II protein and autophagic vacuoles [115]. Likewise, Skog et al. compared the infectivity of different serotypes of adenoviruses in CSCs and non-CSCs sorted out from low-passage brain tumor cells as well as primary glioma cells [116]. They found that infection rates for human adenovirus serotype 16 and chimpanzee adenovirus were similar in both CSC and non-CSC populations. Of note, among dozens of serotypes of human adenoviruses (Ad), Ad5 and Ad2 are the most commonly studied serotypes for their use as vector in gene therapy or as oncolytic candidates. Furthermore, Wakamito et al. isolated CSCs (CD133^+^) from glioblastoma specimen obtained from patients and tested the oncolytic activity of an attenuated herpes simplex virus (HSV), G47Delta [60]. Infection with the virus was found to abrogate the self-renewal ability of the CSCs in vitro and intratumoral injection of the virus prolonged the survival of mice bearing highly invasive intracerebral tumors generated from those CSCs. A more advanced oncolytic HSV encoding two anti-angiogenic proteins endostatin and angiostatin, was shown to effectively kill CSCs derived from glioma patients, in vitro [61]. While the virus was able to kill a majority of the CSCs, a small fraction of CSCs that escaped the virus-mediated killing were found to have lost their self-renewal ability.

### 6.4. Colorectal Cancer

Our group has previously shown that an oncolytic HSV, NV1066, efficiently replicates in tumor-initiating colon cancer cells and causes oncolysis to the same levels as in the unselected bulk colon cancer cells [58]. Consistent with its ability of replicate in and kill the cells in vitro, the virus abrogated growth of CSCs-derived tumors in mice. Another study by Yoo et al., has shown that CSCs (CD133^+^CD44^+^) sorted out from human colon cancer cell lines that were resistant to the drug CPT11, were highly susceptible to an oncolytic vaccinia virus [117]. The virus was found to effectively control growth of spheres, enriched in CSCs, of HT29 (human colon cancer) as well as CT26 (murine colon cancer). The efficacy of the virus in controlling those tumors was further enhanced when combined with 5-fluorouracil [117].

### 6.5. Liver Cancer

As in several other types of cancer, CD133^+^ cells are considered to be CSCs in liver cancer [118]. Several studies have demonstrated that CSCs isolated from established liver cancer cell lines or primary cells from patients are susceptible to oncolytic virotherapy. Zhang et al. studied the oncolytic effect of an adenovirus in which an essential viral gene (E1A) was controlled by a liver cancer-specific promoter sequence (GP73) [62]. In this study, liver cancer cell lines (Huh7 and HepG2) were cultured as 3D-spheres in order to increase the CSC proportions. After verifying that the majority of cells in the spheres were CSCs, the authors compared cytotoxic effect of the virus in cells grown as spheres or as monolayer cultures. They found that the virus was more effective in killing CSCs than non-CSCs, which was shown to be due to high activity of GP73 promoter in CSCs than that in non-CSCs. Another study by Bach et al. used measles virus retargeted to CD133 as a potential oncolytic virus for killing CSCs [63]. The virus robustly killed CD133^+^ cells both in vitro and in mouse models of human liver cancer. Interestingly, while this virus was also found to be effective against CD133^+^ CSCs from glioblastoma and colon cancer, it did not affect CD133^+^ normal hematopoietic cells.

### 6.6. Lung Cancer

Yang et al. constructed an oncolytic adenovirus armed with the apoptosis inducer TRAIL [64]. They tested the cytotoxic ability of the virus in CSCs, enriched through sphere culture of the lung cancer cell line A549. The virus was found to efficiently infect the cells and induce apoptosis in the infected cells. Although CSCs are thought to be resistant to apoptosis, the virus encoding apoptosis-inducer TRAIL was found to be significantly better than the one without TRAIL in killing the CSCs. Furthermore, the virus was able to slow down the growth of xenografts generated from CSCs in mice. Another study by Sagara et al. demonstrated that an oncolytic Coxsackievirus B3 could efficiently kill CSCs isolated from A549 cells [119]. They used the side-population technique based on Hoechst-33342 exclusion to enrich CSCs-like cells from A549. Interestingly, although the killing of CSCs and non-CSCs by the virus was found to be similar, CSCs undergoing EMT were found to be very resistant to the virus. They used TNF-gamma to induce EMT in the CSCs-like cells [119]. In contrast, we have found that induction of EMT has no effect on cytotoxic ability of an oncolytic chimeric poxvirus in A549 cells [120].

### 6.7. Gastric Cancer

CD133^+^ cells are thought be cancer stem cells in gastric cancer. CD133^+^ cells in gastric cancer have been shown to maintain quiescence and are highly resistant to therapeutics that target dividing cells. Yano et al. constructed an oncolytic adenovirus in which the essential genes E1A and E1B were driven by the tumor-specific promoter hTERT [65]. They tested the oncolytic activities of the virus in CSCs established from two gastric cancer cell lines: MKN45 and MKN7. Using 3-dimensional sphere cultures and xenograft tumor models, they elegantly showed that the virus was able to induce cell-cycle mobilization from G_0_-G_1_ to S/G_2_/M phases and ultimately killed the otherwise quiescent CD133^+^ cells. Furthermore, cell-cycle mobilization also made these cells sensitive to chemotherapeutics. Likewise, Zhang et al. constructed an oncolytic adenovirus using hTERT promoter for driving the virus essential gene E1A as well as an apoptosis-inducing gene TRAIL [121]. To test this virus in context of CSCs, the authors established radio-resistant cells from two esophageal cancer cell lines: Seg-1 and TE-2. They isolated stem-like cells based on hoescht-33342 exclusion and verified expression of cancer stem cell markers. Interestingly, the activity of hTERT was found to be significantly higher in the stem-like cells compared to non-stem-like cells, and the cells were efficiently killed by the oncolytic virus. Furthermore, hTERT activity was also found to be significantly elevated in radio-resistant cells compared to the radio-sensitive parental cells, and the radiation-resistant cells were found to be more susceptible to the virus when compared to the parental cancer cells. Overall, the study by Zhang et al. showed that cancer stem-like cells which are resistant to radiation therapy could still be highly susceptible to oncolytic viruses [121].

## 7. Discussions and Conclusions

Since their discovery as a distinct population of cancer cells some two decades ago CSCs have been a subject of intense study, which has resulted in a wealth of knowledge about these cells. CSCs have been isolated from most, if not all, types of cancer and multiple studies have shown that CSCs are inherently resistant to conventional therapies. Given the implications of CSCs in disease relapse and metastasis, novel therapeutics are being sought that could kill not only the differentiated non-CSC cells comprising the bulk of tumors but also the rare CSC population. Although the field of CSCs is progressing at a high pace, there are many questions that remain to be answered in order to fully understand the complexity of CSCs and be able to efficiently target them. For example, most studies have used CSCs enriched in vitro after growing them in serum-free medium as spheroids. However, it is not clear to what extent these spheroid-enriched CSCs represent the original CSCs in tumor in situ. It is likely that CSCs enriched through spheroid culture in serum-free medium may acquire higher survival potency compared to cells that are grown in serum-containing medium. These cells adapted to limited nutrients in vitro are more likely to form tumors when engrafted in immune-deficient mice, popularly known as tumorigenicity assay that is considered the gold standard assay for CSC, regardless of their stemness [122,123]. Likewise, most studies have used antibodies-based cell-sorting technique to separate cells into CSCs and non-CSCs to test the responsiveness of the two separate populations of cells to therapeutics. However, it is not clear if antibodies bound to cell-surface has any effect on those cells, especially on the stemness of the cells. Furthermore, the side-population technique based on Hoechst staining is also a commonly used technique to separate CSC and non-CSCs and compare their response to therapeutics. Hoechst may also affect the survival of cells, which seems to have been overlooked in most studies. Despite the current limitations in the field, it is well established that compared to differentiated cells, cells with CSC properties, even when heterogeneous, are more resistant to traditional therapies.

Resistance to traditional therapies is mainly because of the ability of CSCs to maintain quiescence and their resilience to apoptosis. Most chemotherapeutics rely on inducing apoptosis to kill cancer cells. It is, therefore, unsurprising that CSCs could escape these therapeutics. Likewise, radiation therapy that relies on killing rapidly dividing cells by inducing DNA damages, fails to kill CSCs as they can maintain quiescence for a long time and they also have higher ability to repair DNA damage. Unlike radiation and chemotherapeutics, many oncolytic viruses including vaccinia virus, adenovirus, HSV, and reovirus can infect both quiescent and dividing cells and replicate efficiently in those cells. Consequently, most oncolytic viruses tested against CSCs have been found to have more or less similar efficacy in killing CSCs and non-CSCs. However, CSCs from different types of malignancies may not be equally susceptible to an oncolytic virus. Alternatively, CSCs from the same malignancy may not be susceptible to all types of oncolytic virus. Therefore, it would be simplistic to generalize oncolytic viruses as cure-all for all malignancies. Nevertheless, oncolytic viruses hold promise for better treatment of cancer. While several preclinical studies have shown that oncolytic virus as a monotherapy may be effective against some malignancies, it would be logical to combine oncolytic virus with traditional therapies to achieve greater therapeutic benefits. Given the fact that oncolytic viruses and traditional therapies exert their anti-tumor effect through different mechanisms, one would expect to achieve additive, if not synergistic, anti-tumor effect from combination therapies. Indeed, several studies have shown that combination of oncolytic virus with chemotherapy or radiation therapy results in synergistic anti-tumor effect in animal models [124,125,126,127]. Additionally, transgenes ranging from toxic genes for direct killing of cancer cells to immune-stimulatory genes for activation of anti-tumor immunity could be inserted into oncolytic viruses to further increase the overall efficacy of oncolytic viruses.

One major concern in the use of oncolytic virus for killing CSCs is that CSCs have many properties in common with normal stem cells. Therefore, oncolytic viruses may kill CSCs and normal stem cells to similar levels. However, several studies have shown that despite similarities between normal stem cells and CSCs, oncolytic viruses specifically kill CSCs while leaving normal stem cells unharmed. Furthermore, most of the animal studies performed with oncolytic viruses have found oncolytic viruses to be very safe. Also, oncolytic viruses have shown excellent safety profiles in clinical trials. Taken together, oncolytic viruses hold great promise for better treatment of cancer as they can kill both differentiated cancer cells as well as CSCs and minimize the risk of disease relapse.

## Figures and Tables

**Table 1:** Examples of oncolytic viruses (OVs) that are effective against CSCs of different origins.

Cancer Type	OV	CSC Source	CSCs Susceptible to OV?	OV Replicates in CSC?	Comments	Reference
CML	Ad	Imatinib-resistant CML patients	Yes	NA	Inhibition of colony formation in vitro and elimination of xenografts in mice	[52]
AML	MYXV	AML patients	Yes	Yes	Prior infection of tumor cells with virus prevented engraftment in 90% of recipient mice compared to mock-infected cells	[53]
Breast	Ad	Pleural effusion from breast cancer patients	Yes	NA	Effective killing of CSCs in vitro; prior infection of CSCs prevented formation of xenografts; anti-tumor effect against CSCs derived tumors in mice	[54]
Breast	Ad	Pleural effusion from breast cancer patients	Yes	NA	Eradication of CSCs in vitro; anti-tumor effect against CSCs derived tumors in mice	[55]
Breast	HSV	Mammospheres generated from breast cancer cell lines	Yes	NA	Highly toxic to CSCs in vitro and effective against CSC-derived xenografts in mice	[56]
Breast	Reo	Human breast cancer patients	Yes	NA	Ras expression, a determinant of reovirus oncolysis, was similar in CSCs and non-CSCs; similar killing of CSC and non-CSCs both in vitro and in xenografts	[57]
Colon	HSV	Tumorspheres generated from HCT8 cells	Yes	Yes	Highly toxic to Akt overexpressing CSCs; effective against CSC-derived xenografts in mice	[58]
Glioblastoma	Ad	Glioblastoma patients	Yes	Yes	CSCs over-expressed Ad receptor (CAR) and were highly susceptible to the virus in vitro; significant anti-tumor effect against CSC-derived xenograft	[59]
Glioblastoma	HSV	Glioblastoma specimen from human patients	Yes	Yes	Effective killing of CSCs in vitro and significant anti-tumor effect against xenografts in mice	[60]
Glioblastoma	HSV	Glioblastoma specimen from human patients	Yes	NA	Effective oncolysis of CSC in vitro and anti-tumor effect against CSC-derived xenografts in mice	[61]
Liver	Ad	Liver cancer cell lines	Yes	NA	Highly toxic to CSCs both in vitro and in xenograft models	[62]
Liver	Measles	Surgical specimen from liver cancer patients	Yes	NA	CD133-targeted OV selectively killed CD133^+^ CSCs and prolonged survival of mice bearing orthotopic xenografts	[63]
Lung	Ad	Tumorspheres generated from A549 cells	Yes	NA	TRAIL-encoding OV was toxic to CSCs in vitro and showed significant anti-tumor effect against CSCs derived xenografts in mice	[64]
Gastric	Ad	Human castric cancer cell lines	Yes	Yes	The virus first-induced cell-cycle mobilization from G0-G1 to S/G2/M in CSCs and then killed them; OV also sensitized those cells to chemotherapies	[65]

Notes: CML, chronic myeloid leukemia; AML, acute myeloid leukemia; Ad, adenovirus; HSV, herpes simplex virus 1; MYXV, myxoma virus; CAR, NA, not assessed; coxsackivirus adenovirus receptor; TRAIL, TNF-related apoptosis-inducing ligand.
